# The Perceptions of Healthcare Professionals Regarding Violence Against Women in Ecuador: A Qualitative Study

**DOI:** 10.3390/healthcare14091146

**Published:** 2026-04-24

**Authors:** Anabel Fernández-Vargas, Otilia Vanessa Cordero-Ahiman, Diana Patricia Vanegas-Coveña, Andrea C. Valencia-Altamirano, Juan José González-Gerez, Cayetano Fernández-Sola, José Manuel Hernández-Padilla

**Affiliations:** 1 International Doctoral School, University of Almeria, 04120 Almeria, Spain; afv487@inlumine.ual.es; 2 Critical Care Unit, Hospital Universitario Torrecárdenas, 04009 Almeria, Spain; 3 Department of Economics, Business and Sustainable Development, University of Cuenca, Cuenca 010201, Ecuador; otilia.cordero@ucuenca.edu.ec; 4 Faculty of Medical Sciences, University of Cuenca, Cuenca 010107, Ecuador; diana.vanegas@ucuenca.edu.ec; 5 Facultad de Ciencias Sociales y Humanas, Universidad Central del Ecuador, Quito 170129, Ecuador; acvalencia@uce.edu.ec; 6 Department of Nursing, Physiotherapy and Medicine, University of Almeria, 04120 Almeria, Spain; juanjogg@ual.es (J.J.G.-G.); j.hernandez-padilla@ual.es (J.M.H.-P.); 7 Facultad de Ciencias de la Salud, Universidad Autónoma de Chile, Santiago 7500000, Chile

**Keywords:** abuse, qualitative study, healthcare professional, violence against women, gender-based violence

## Abstract

**Highlights:**

**What are the main findings?**

- Violence against women can be expressed in subtle ways.
- There is a social dimension to violence against women that tends to normalize certain violent practices.

**What are the implications of the main findings?**

- Healthcare professionals can develop strategies to prevent violence against women by identifying it at an early stage.
- Healthcare professionals may offer multidisciplinary care and play an active role in sex education.

**Abstract:**

**Objective:** The aim of this study was to explore how healthcare professionals in the Republic of Ecuador perceive violence against women, its underlying social determinants, and their role in prevention and response within the healthcare setting. **Methodology:** A qualitative descriptive study was conducted using purposive sampling. Ten healthcare professionals with experience in managing cases of violence against women participated. Data were collected through semi-structured interviews and analysed using Braun and Clarke’s reflective thematic analysis. The ATLAS.ti software was used throughout the analysis process. **Results:** The participants emphasised the normalisation of microaggressions, institutional shortcomings in prevention systems, and the need for gender-sensitive professional training. Three main themes emerged from the data analysis: (1) the characteristics and identification of violence against women, (2) the social dimensions of violence against women, and (3) combating violence against women in clinical and educational settings. **Conclusions:** The healthcare professionals highlighted the need to recognise normalised and less visible forms of violence reflected in everyday attitudes and behaviours. They emphasised the importance of early identification, multidisciplinary care and sex education as preventive strategies. The social context and deep-rooted power dynamics favour the perpetuation of violence against women. Healthcare professionals can play an important role in the prevention of violence against women by improving care for survivors, identifying areas for improvement within existing prevention systems, and promoting sex education.

## 1. Introduction

Worldwide, approximately 30% of women have experienced physical and/or sexual violence, and 38% of femicides are committed by an intimate partner [1]. In Ecuador, between January and September 2024, at least 180 women, adolescents and girls were killed as a result of violence against women [2]. These figures highlight that violence against women constitutes both a major public health concern and a serious violation of human rights [3]. Healthcare professionals play a crucial role in identifying, preventing, managing, and supporting the recovery of victims of violence against women [4]. However, healthcare workers require clear, specific training and guidance to provide effective interventions and appropriate care for victims [5].

Violence against women is defined as any act of gender-based violence that may result in physical, sexual or psychological harm to women, including threats of such acts, coercion or arbitrary deprivation of freedom, whether in public or private life [1]. Some of the physical effects of violence against women include trauma, wounds, burns, bruises, bleeding, haematomas, urinary tract infections, chronic pelvic pain and headaches [6]. On a cognitive level, violence can affect the brain, causing alterations in executive functions, working memory and processing speed [7]. Psychological effects include depression, anxiety, post-traumatic stress, and low self-esteem [8]. The consequences for sexual and reproductive health include loss of sexual desire, menstrual disorders, sexually transmitted diseases, vaginal bleeding, dyspareunia, unwanted pregnancy, and potential miscarriage [9].

To protect women from any violent action or behaviour, Ecuador created the Comprehensive Organic Law to Prevent and Eradicate Violence against Women [10]. However, despite the solid legal framework, there is still a significant discrepancy between the law and its practical application [11]. Despite its robust legal framework, the implementation of Ecuador’s Comprehensive Organic Law hindered by critical barriers, including a lack of granular data, low reporting rates, and prolonged judicial processes that often result in impunity. These structural challenges are further compounded by a patriarchal culture that prioritizes family stability over victim safety, as well as a chronic lack of institutional resources [12]. In-depth qualitative studies on how this specialized system functions in practice remain scarce; thus, there is a priority to examine whether the system truly responds to the lived realities and needs of survivors, rather than merely existing as a formal set of rules [13]. Healthcare professionals can play an important role in both the prevention of violence against women and the recovery of the victims [14]. Their role depends largely on law enforcement systems to provide justice and health care to victims [15]. In many contexts, healthcare professionals report insufficient training in identifying and responding to violence against women [16], even though it is known that healthcare professionals with more extensive knowledge about violence against women are more effective in supporting victims of violence against women [17].

Healthcare professionals should facilitate disclosure, offer emotional support, refer victims to social services, and monitor them throughout the process of responding to violence against women [18]. However, a lack of knowledge about how to respond to situations of violence against women leads professionals to avoid addressing the issue directly, even when there is evidence of abuse [19]. In order for healthcare professionals to provide comprehensive health care from a gender perspective, it is necessary to learn about the experiences of women who have survived violence against women. This includes knowing how to identify and respond to violence and understanding how these women experience the various stages involved. Nevertheless, healthcare professionals who have contact with victims show high levels of exhaustion in response to chronic emotional stress caused by workload, lack of expertise and skills on how to act, uncertainty, lack of communication skills, and lack of support [20]. There are studies on the effects of violence against women, its causes [21] and legal aspects [22]. Although previous studies have examined the health consequences of violence against women and legal frameworks in Ecuador, there is a lack of research on how healthcare professionals conceptualise, interpret and operationalise their role in prevention and response. Understanding these perceptions is essential, as they shape clinical decision-making, detection practices and institutional responses.

The objective of the study was to explore how healthcare professionals in the Republic of Ecuador perceive violence against women, its underlying social determinants, and their role in prevention and response within the healthcare setting.

## 2. Materials and Methods

### 2.1. Design

A qualitative descriptive design grounded in an interpretivist paradigm was adopted to explore participants’ subjective understandings of violence against women. This approach allowed the researchers to closely engage with the data, capturing participants’ perceptions, experiences and opinions in depth. It focuses on how participants perceive, interpret, and experience a phenomenon [23]. This report was prepared in accordance with the COREQ guidelines [24] included in the Supplementary Materials (Table S1).

### 2.2. Participants and Context

The study was conducted as part of an international cooperation project for the prevention of gender-based violence between the University of Almería, Spain, and the Universities of the Americas, Azuay and Cuenca, Ecuador. Participants were recruited using purposive sampling, with the aim of achieving maximum diversity in terms of professional background and work experience. The researchers selected the sample based on the relevance of their work experience to the objective of the study. The inclusion criteria were: (1) being a healthcare professional, and (2) having work experience in cases of violence against women. The exclusion criteria were: (1) refusing to participate in the study. A total of 14 healthcare professionals were invited to participate by email, of whom 4 declined due to scheduling issues. Therefore, the study included a total of 10 healthcare professionals with a mean age of 43.3 years (SD = 9.9). Table 1 shows the sociodemographic characteristics of the participants (Table 1).

### 2.3. Data Collection

Semi-structured interviews progressed from general topics to more specific issues, enabling the extrapolation of relevant information to address the study objectives. The interviews lasted an average of 45 min and were conducted face-to-face at the participating universities between September 2023 and January 2024. The interviews were conducted by three women researchers with expertise in violence against women and qualitative data collection (DPVC, OVCA, ACVA). A single interview was conducted with each participant in Spanish, so no repeat interviews were conducted. Data saturation was determined when no new themes or subthemes emerged from the latest interviews. The sample size of 10 participants was considered sufficient due to their high information power, characterized by their extensive specialized experience and the analytical depth of the semi-structured interviews, which provided a comprehensive understanding of the structural and clinical dimensions of violence in this specific context. The researchers used an interview protocol (Table 2) that was tested beforehand. The interviews were recorded and transcribed verbatim in Spanish, and notes were taken on non-verbal elements of communication.

### 2.4. Data Analysis

The data obtained in the interviews were transcribed verbatim and notes were taken on non-verbal elements. The data were analysed using Braun and Clarke’s reflective thematic analysis method [25], which consists of six steps:

(a) Familiarisation with the data was achieved through repeated reading of the interview transcripts to ensure immersion in the data, with field notes integrated throughout the analytic process. Familiarization memos were recorded using the memo function in ATLAS.ti 25 (ATLAS.ti Scientific Software Development GmbH, Berlin, Germany).

(b) Systematic data coding: Data coding was a collaborative and iterative process involving three independent researchers. To ensure rigour and minimize bias, the team held regular meetings to reach a consensus on code definitions and the selection of representative quotes. While ATLAS.ti facilitated data management, the analysis was driven by the team’s reflexive and collective interpretation.

(c) Generation of initial themes from coded and collated data: codes that shared similar ideas or concepts were grouped into sub-themes and themes. During this phase, ATLAS.ti networks were used as a visual aid to map the relationships between categories and refine the analytical structure.

(d) Development and review of themes: the sub-themes created were verified to ensure they were consistent with the codes generated and the quotes coded from the interview content. Two researchers reviewed the sub-themes to verify that they addressed the study objective.

(e) Refining, defining, and naming themes: The sub-themes generated were grouped into main themes, and final names were created for each theme and sub-theme. Themes and sub-themes were defined and explained.

(f) Drafting the report: for the preparation of this research report the most representative or eloquent quotes were extracted to provide evidence for the themes and sub-themes. Finally, the themes were linked to each other and to the literature review. In this phase, a concept map was built to illustrate the explanation of the phenomenon.

### 2.5. Ethical Considerations

The study was conducted in accordance with the ethical principles of the Declaration of Helsinki [26]. Permission was obtained from the ethics and research committee of the Department of Nursing, Physiotherapy and Medicine at the University of Almería (EFM 141/2021). All participants were informed of the methodology and objective of the study. They were made aware of their right to withdraw from the study at any time, as well as the possibility of declining to answer questions. Each participant signed an informed consent form before the interview began and were given a transcript of their interview after it was conducted. In addition, the participants were informed about data confidentiality in accordance with the Data Protection Act [27].

### 2.6. Rigour

The study followed Lincoln and Guba’s quality criteria for qualitative research at every stage [28]. The credibility of the study was ensured through several factors. First, the sample consisted of healthcare professionals who actively work with victims of violence against women. Second, data were collected through interviews, and third, the analysis was reviewed by two researchers to verify the findings. To ensure transferability, the working method, participants and context in which the data were collected were described in detail. Dependability was supported by a detailed description of the methodology section. Researchers reflected on their professional backgrounds and prior experience in gender-based violence to critically examine how these positions could shape data interpretation. Lastly, confirmability was ensured through data analysis triangulation and by verifying that each participant’s perspectives were accurately reflected in the transcription of their interviews and the results of analysis.

## 3. Results

Three main themes emerged from the analysis of the data, which characterise healthcare professionals’ perceptions of violence against women (Table 3).

### 3.1. Theme 1. The Characteristics and Identification of Violence Against Women

This theme addresses the key characteristics of violence against women. Identifying such violence may facilitate early intervention by healthcare professionals and contribute to improved quality of care for victims. Three sub-themes emerged from this theme.

#### 3.1.1. Subtheme 1. Normalisation of Microaggressions as Structural Violence

According to the participants’ perceptions, certain forms of violence against women go unnoticed in society, with many gender-based microaggressions being normalised. Similarly, several healthcare professionals highlighted the need to make violence visible in all its forms.

“*We need to change our mindset; it’s not a gender-based microaggression, it’s violence! Catcalling on the street is normalised violence!”*

(I-1)

Healthcare professionals identified several non-specific signs and symptoms that may help to identify victims of violence against women. Awareness of these warning signs is essential to determine whether care and protection protocols should be activated. The signs described were mainly negative in nature and included reduced motivation and vitality, as well as insomnia, loss of appetite and apathy.

*“Physically, it can be insomnia, loss of appetite, distress, anxiety (…) and some symptoms and signs that do not correspond to any particular condition, so you know that everything hurts them and that you always have to find out what is going on in their intimate partner relationship.”*

(I-5)

Furthermore, the participants highlighted that there are different types of violence against women. They specified that victims must be cared for in all areas, including the biopsychosocial sphere, and that inquiries must be conducted to detect and confirm cases of violence against women.

*“Physical violence is reported either by the victim themselves or by witnesses when they hear screams or something, but other types of violence are completely covered up; they do not correspond to reality.”*

(I-2)

These narratives reveal an implicit diagnostic model where professionals construct the recognition of violence through an epistemic shift: they reclassify social ‘normality’ (microaggressions) into systemic violence, and interpret vague psychosomatic symptoms (insomnia, generalized pain) as clinical proxies for abuse. Consequently, the epistemic recognition of violence relies on the practitioner’s ability to ‘read between the lines’ of symptoms that do not correspond to organic conditions, placing the professional in the role of an interpreter of silenced social suffering.

#### 3.1.2. Subtheme 2. At the Bottom of the Iceberg: Identifying Implicit Violence

The participants referred to the common metaphor of violence against women as an iceberg. They spoke of an implicit or subtle level of violence, consisting of sexist humour, sexist comments, gender roles, etc. This constitutes the foundation upon which other more explicit forms of violence against women can develop.

*“…at the bottom of the iceberg there are discriminatory behaviours that later become more serious or that do not progress beyond that stage, (…). These include sexist jokes, comments, situations in which women are labelled, ways of implying a certain inferiority or denying a right.”*

(I-7)

Narratives surrounding violent or discriminatory behaviour contribute to its normalisation when the victims are women or individuals who do not conform to heteronormative norms. These narratives play a powerful role in legitimising behaviours that are experienced as violent by those who suffer them.

*“…the way stories are told, how violent acts are normalised when they are directed at certain people, but the narrative has a significant influence on how the message is communicated, so that is also being normalised.”*

(I-3)

Healthcare professionals believe that subtle cases of violence against women that go unnoticed by society are related to cultural factors. For this reason, the participants suggested that discriminatory and/or violent attitudes towards women may be unconsciously transmitted through education.

*“…it is likely that these elements of violence are being perpetuated in educational settings without people being aware of it.”*

(I-8)

Participants noted that their accumulated clinical experience has refined their ability to identify victims of violence. However, they emphasized that professional competence also involves the deliberate decision to avoid revictimization. This implies recognizing situations where clinical or legal evidence is already sufficient, thereby making it unnecessary—and ethically counterproductive—to force the victim to relive the trauma through repetitive disclosure.

*“With practice, one can detect a victim almost immediately. If it is a woman who comes in with physical evidence and has been referred, for example, by a legal clinic, it is no longer necessary to ask her to retell her story.”*

(I-10)

One participant noted that, while efforts have been made to increase the visibility of gender-based violence, invisible forms remain prevalent and underreported. This suggests that healthcare professionals must remain vigilant toward these non-evident manifestations that profoundly affect victims. One professional proposed a specific clinical strategy to uncover these hidden dynamics:

*“… I really like, as a diagnostic tool, the interview where we can construct a sort of life history… addressing small things, like how social media is managed, what kind of jokes or comments the husband makes with his friends…, how her work is valued, or how child-rearing is handled (…) addressing household chores, which are a breeding ground for sexism; these are practices that (…) a psychologist doesn’t always ask about, but perhaps it would be a good idea to do so”*

(I-1)

These examples demonstrate that awareness of implicit violence is not only fundamental for theoretical understanding but must also function as an active component of clinical practice. By integrating a biographical and micro-sociological lens into the consultation, professionals can transform routine inquiries into tools for identifying structural oppression, ultimately fostering a safer and more empowering environment for victims.

### 3.2. Theme 2. The Social Dimensions of Violence Against Women

The violence experienced by victims is shaped by the social context in which they live. In this regard, healthcare professionals highlighted the need for broader societal change to address social challenges such as exclusion and inequality. This theme comprises three subthemes.

#### 3.2.1. Subtheme 1. The Social Factors Underpinning the Persistence of Violence Against Women

From the participants’ perspective, situations of inequality experienced in society are strongly shaped by cultural factors, religion and social traditions. However, they noted that such inequalities are becoming increasingly visible in contemporary social contexts.

*“(…) I think that, in the local context, there are traditions, the same religion, cultural aspects that lead to patterns being followed that are culturally assigned to men or women. This makes it a sexist society, one of customs, of respecting family traditions and a series of situations that, incredibly, [amazingly] are still present in the 21st century.”*

(I-4)

Furthermore, the participants considered that social traditions assign roles to individuals based on gender and social context. When these roles are challenged or changed, tensions and discrepancies may emerge within society. However, the healthcare professionals emphasised that these stereotypical roles are deeply embedded in social structures and are therefore difficult to dismantle.

*“… from the moment we are born into families, we are assigned gender roles, and the moment those roles are broken, conflicts begin to arise in society, where the possibility of breaking that rigid gender structure is not accepted.”*

(I-1)

Furthermore, the participants interpreted religion as a conditioning factor with deep roots in society. They argued that it can reinforce gender stereotypes that contribute to inequality and, in addition, perpetuate sexist beliefs that may lead to violence against women.

*“Religion is extremely sexist, hence gender stereotypes and double standards regarding sexuality,* i.e.*, aspects that serve a practical purpose but ultimately result in inequality between men and women, which in some cases leads to experiences of violence.”*

(I-7)

The participants’ narratives reveal a socio-cultural scaffolding where violence functions as a regulatory mechanism. They perceive a temporal paradox: despite 21st-century progress, rigid structures like religion and tradition act as stabilizing forces for inequality. In this context, challenging traditional gender roles is interpreted as a breach of a social contract that the system—through violence or institutional silence—seeks to punish or normalize under the guise of ‘customs’.

#### 3.2.2. Subtheme 2. Power Dynamics That Encourage Violence Against Women

Violence against women is a public health issue that health and social services cannot overlook. The participants argued that women frequently face difficulties imposed by societal structures and norms. As a result, women are often required to resist social violence on an ongoing basis, frequently without sufficient institutional or social support.

*“Yes, it is a public health issue because women (…), are in a constant state of resistance. At some point, it raises the question: how long can I keep resisting? When will I find empathy from others to end this resistance and start building something new?”*

(I-3)

The participants noted that certain powerful groups enjoy greater privileges and promote inequality between men and women while gaining increasing social influence. This can lead to social inequality, thus underscoring the need to challenge these power dynamics.

*“The way society is organised, half of its members enjoy privileges that they do not want to give up. On the other hand, these anti-rights groups are gaining ground and questioning women’s initiatives to achieve equality.”*

(I-2)

In addition, the participants emphasised that the implementation of policies in favour of women can lead society to perceive equality as an opportunity rather than as a fundamental human right. They argued that it is not a question of opportunities, since equality is a right for both men and women, as well minority groups.

*“(…) A right is not an opportunity! A right is something that belongs to us, it is not something that is given! It is something that no one should take away from us, because it is a right!”*

(I-9)

The participants perceive that, although Ecuadorian law offers a solid legal framework, its application faces serious practical challenges. These include a lack of resources, disbelief toward victims, and the need for greater awareness and education regarding gender-based violence.

*“…anyone who reads the law … says, ‘Wow, what an advanced country!’ But when that law hits reality, it’s not all it’s cracked up to be. First, … there’s still doubt about a woman’s word; …those who are raped, for example, they aren’t believed from the get-go!”*

(I-2)

*“…once you file a report, you must have evidence, you need witnesses, and in the end, it turns into a confrontation—her word (the woman’s) against her aggressor’s. He can simply say it was consensual, or he can deny it; and since there’s no evidence, no witnesses, and no proof, the toll on the victim is just terrible.”*

(P-1)

The quote demonstrates that disbelief toward the report is a serious obstacle to the application of the law, however advanced it may be. Furthermore, the lack of specialized personnel and political will to effectively implement the law translates into inadequate care for victims—such as the lack of reagents for forensic testing—which can lead to the loss of crucial evidence.

*“There are improvements! (…) but not long ago I had the case of a woman who was raped; when she got to the prosecutor’s office for a forensic assessment, they didn’t have the reagents, or the supplies needed to do it. (…) They never analysed the clothes for DNA testing. Plus, with institutions that are perpetually in crisis and have nothing—they lack psychologists, they lack professionals—no, it’s not being fulfilled (The law), no…”*

(I-6)

#### 3.2.3. Subtheme 3. Social Approaches to Combating Violence Against Women

When victims of violence against women seek healthcare services, healthcare professionals are the first to attend to them. Therefore, training healthcare professionals to identify cases of violence against women is one of the main strategies for detecting victims. Access to health-related knowledge and skills contributes to the improvement of clinical practice and increases confidence among healthcare professionals. However, the participants perceived a lack of capacity to obtain the information necessary to detect cases of violence against women.

*“We are unable to understand. What is hurting people’s souls and what is happening in society? I believe that our health services do not diagnose or detect much in terms of violence against women, violence against diversity, or the violation of people’s rights.”*

(I-5)

In some cases, the healthcare professionals felt that protocols for responding to violence against women were not being followed. In fact, they commented on the need for a reviewing body to ensure that policies were being implemented correctly and that decisions were being made appropriately in cases of violence against women.

*“We must remain vigilant. It would be interesting to have an observer monitoring compliance with these elements to see if they are actually being implemented and, (…) to what extent there is political pressure (…) from which you can make decisions and where your decisions do not have to be watered down.”*

(I-3)

According to the participants, resources for victims of violence against women function as strategies to promote their well-being and quality of life. They viewed subsidies as an opportunity to escape and confront situations of violence. In this regard, the participants emphasised the importance of implementing strategies focused on providing safety and guaranteeing the victims’ individual rights.

*“There are those who think that the solution probably lies in policies, that is, increasing policies where women, for example, are empowered, receive more financial aid, and there are more public resources, which will apparently improve the situation.”*

(I-4)

The participants conceptualize socio-economic empowerment as a critical protective factor, viewing financial resources not merely as aid, but as the material foundation for autonomy necessary to escape violent environments.

Professionals conceptualize their duty as an exercise in clinical hermeneutics, where the focus shifts from treating isolated injuries to decoding the ‘unspoken’ narratives of trauma. By emphasizing the need for interdisciplinary teams and specialized training, they advocate for a systemic social approach rather than a purely medicalized one. This construction suggests that combating violence requires the professional to act as a social mediator, capable of overcoming institutional resistance and addressing the biopsychosocial complexities that silence victims. Consequently, high-quality care is redefined as a coordinated social intervention that integrates the victim’s subjective experience into a broader framework of protection and advocacy.

### 3.3. Theme 3. Combating Violence Against Women in Clinical and Educational Settings

This theme highlights the role that healthcare professionals should play in addressing violence against women, while also drawing attention to shortcomings within the system that need to be identified and addressed. To combat violence against women, the participants agreed that education is a key area for intervention with future generations.

#### 3.3.1. Subtheme 1. The Duty of Professionals in Addressing Violence Against Women

The care provided to victims of violence against women should be tailored to their individual experiences. The healthcare professionals also emphasised the need for a comprehensive approach consistent with each woman’s situation.

*“Addressing violence against women requires a comprehensive approach, because it depends on the type of violence. All types are important, but obviously some situations are more complex than others. ‘Yelling at someone is not the same as hitting them!”*

(I-8)

When caring for victims of violence against women, healthcare professionals must consider the psychological process the victim is undergoing. The participants highlighted the importance of taking into account the language used by victims and emphasised that care should incorporate their needs from a multidisciplinary perspective.

*“When it comes to mental health, I think it’s essential to pay attention to the narratives… and what the person says, even what’s difficult to say, (…). What’s difficult to say could be a symptom of an underlying situation, right? Avoiding it, not saying it directly.”*

(I-9)

Various limitations complicate the care process for victims of violence against women. To provide high-quality care, healthcare professionals require greater training and specialisation in this area. In addition, interdisciplinary teams need to be strengthened to enable coordinated work and facilitate the provision of care to victims.

*“Some more training is needed, because every institution should have a specialised team that knows how to address these issues, precisely because of the reaction they provoke and the resistance that exists, in order to have solid arguments on which to base any defence…”*

(I-2)

Participants identified significant shortcomings in institutional capacity, particularly regarding the need for specialized training. These systemic barriers to care are perceived as forms of institutional resistance that hinder professionals’ ability to firmly defend victims’ rights.

By emphasizing experiences that are hard to articulate, practitioners advocate for a model of care that integrates psychological depth with systemic advocacy. This implies that combating violence is not merely a medical task but a collective professional responsibility to build solid, argument-based defences within the healthcare system to protect victims from further social or institutional vulnerability

#### 3.3.2. Subtheme 2. Identifying Systemic Failures in the Eradication of Violence Against Women

The society in which we live is marked by discrimination based on race, gender, religion, sexual orientation, gender identity and illness. In situations of violence against women, the first step is to validate the victim’s testimony, which may be influenced by characteristics attributed to the victim, such as being white or black, heterosexual or homosexual, or educated or uneducated.

*“Firstly, (…), a woman who does not have the means to make the problem visible is less likely to be believed, and by means, I mean a woman who has an education, because if she is studying, she may not have the support to do so, to give her the value she deserves. This implies that patriarchal culture values education and not the source of the problem.”*

(I-3)

Furthermore, according to the healthcare professionals’ experience, victims of violence against women are affected at a biopsychosocial level. Despite this awareness, the care provided by the system focuses mainly on the victim’s physical condition. The participants perceived that there was a lack of resources to address victims’ psychological and social needs.

*“Behind physical violence there is a whole imaginary world, a deeper meaning. We need to work on this, at least in terms of mental health. First, we need to work on the implications, because we say, ‘The first thing is to separate from the partner,’ but how can we guarantee that leaving her partner will be good for that woman emotionally? Because often a woman’s partner is incredibly important to her, so it’s not that simple! It’s not that easy.”*

(I-7)

In addition, the healthcare professionals reported a lack of financial resources to prevent, promote and encourage change among victims of violence against women. They also highlighted the lack of resources to address needs associated with legal and healthcare processes. Furthermore, in many cases victims are unable to bear the costs resulting from economic violence.

*“The system is imperfect; it lacks personnel, specialisation, resources and (…), political will. For example, in 2019, the budget for law enforcement was reduced by more than 80%. (…) Ecuador’s law is fantastic, seeking to empower victims and make them autonomous, but there are no resources to undertake initiatives that would enable them to move forward.”*

(I-2)

#### 3.3.3. Subtheme 3. Promoting Sex Education

The participants expressed several moral concerns regarding education. They indicated that it has been shaped by social thinking, policies and traditions characterised as sexist and patriarchal, thus limiting the diversity of perspectives represented in educational settings. The healthcare professionals also argued that sex education in schools tends to be oriented towards heteronormativity, without adequately addressing diverse sexual orientations and identities.

*“From its inception, school was created to homogenise (…). Furthermore, school is linked to the Catholic religion, so I continue to stress to students that the changes we need to make in school are countless!”*

(I-4)

The participants reported that education should not be limited to schools, and that they themselves have a responsibility to engage in ongoing training and self-education in order to promote a different social perspective. They also stated that all healthcare professionals should take part in the process of social change through sex education.

*“…I do believe that education plays a fundamental role, the training of professionals in general; psychologists, communicators, educators, doctors, that is, only when the teacher who is responsible for training other professionals has a different vision.”*

(I-10)

Furthermore, the healthcare professionals commented that a new era of social change is beginning. The younger generations are showing a more open-minded attitude towards social change and greater respect for sexuality. However, there is still much work to be done, as social barriers prevent people from expressing their feelings without prejudice.

*“I want to do this, but society won’t let me, no! Or I have certain impulses within my sexuality, the way I want to express myself, feel and act with my partner, but I’m afraid they’ll leave me. What will they think of me? I feel like I’m going against traditional family values. I think the discourse is starting to sink in, yes! It’s starting to shape our minds.”*

(I-7)

Participants conceptualize the educational system as a homogenizing apparatus that reinforces patriarchal and heteronormative hierarchies. Their narratives reveal a structural tension between the school’s traditional role—preserving religious morality—and the urgent need for a vision that embraces diversity. Interpretively, professionals view education as a territory of struggle, arguing that subverting institutionalized sexism requires ‘re-training the trainers.’ Thus, education is framed as both the site where inequality is replicated and the primary vehicle for its deconstruction.

**Figure 1:**
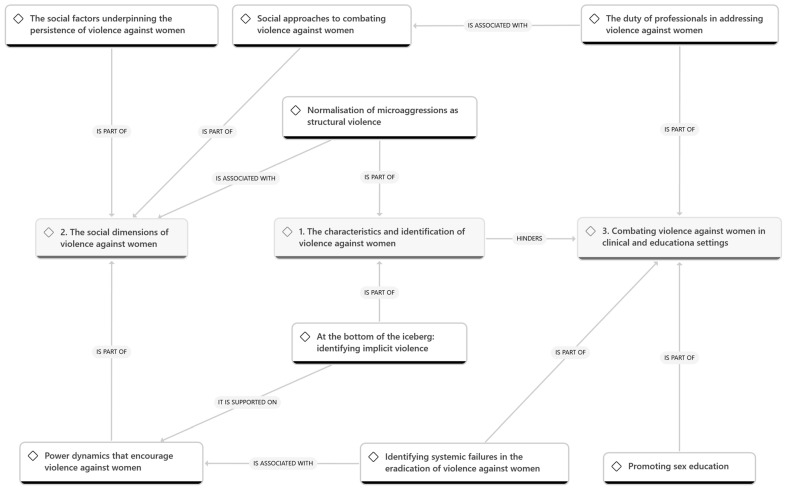
Conceptual map.

At a pragmatic/interpretative level of analysis, a conceptual map (Figure 1) was developed to represent the relationships between themes and provide a deeper explanation of the phenomenon.

The results suggest that the normalization of microaggressions and the persistence of implicit violence (Theme 1) are not isolated phenomena but are deeply rooted in structural gender asymmetries and power dynamics sustained by traditional religious and social socialization (Theme 2). This cultural scaffolding creates a “breeding ground” for violence that complicates its identification in clinical settings, as both victims and professionals often operate within the same normalized framework. Consequently, combating this violence requires a shift from a purely medicalized gaze toward a systemic social approach. This involves redefining the professional’s duty not only as an expert in detection but as an advocate for the victim’s autonomy, intentionally working to avoid revictimization despite existing institutional capacity gaps. Ultimately, the educational and clinical apparatuses are framed as the primary sites for subverting these hierarchies, suggesting that only through specialized training and the promotion of transformative education (Theme 3) can the cycle of institutionalized violence be effectively dismantled.

## 4. Discussion

The objective of the study was to explore the perceptions of healthcare professionals regarding violence against women and its prevention in the Republic of Ecuador. The study design provided a comprehensive description of how violence against women is perceived, interpreted and experienced from a personal, social and care perspective [21]. The data analysis identified three central themes: the characteristics and identification of violence against women, the social dimensions of violence against women, and combating violence against women in clinical and educational settings.

The participants associated experiences of violence against women with a range of signs and symptoms that indicate abuse. At a psychological level, the abuser’s behaviour may include verbal aggression or degradation, such as threats, and may lead to symptoms of depression and anxiety, as well as feelings of shame and self-blame [29]. There is also physical violence, which involves forced physical contact between partners, and may result in injury or functional impairment [6]. These forms of violence can produce serious consequences across multiple areas of the victim’s life [30]. Abuse and harassment are frequently linked to health problems that contribute to functional limitations and dependence, while also associated with feelings of humiliation and insecurity [31].

The participants highlighted the need for a shift in societal attitudes. They referred specifically to a lack of awareness surrounding violence against women, linked to the patriarchal pact embedded within social culture [32]. Power relations, perpetuated both in society and within families, assign a lower value to women through patriarchal dominance, thereby perpetuating violence against women [33]. This is also associated with systematic silence, understood as the tendency to omit, invalidate, reject or deny that violence against women has occurred [34].

In terms of the healthcare context, the healthcare professionals frequently reported that the care provided to victims of violence against women is insufficient. Action plans must ensure adequate support to prevent victims from being overlooked [35]. Addressing this issue requires an approach that eliminates barriers related to false beliefs, ignorance, prejudice, social norms and patriarchal culture, while incorporating gender-sensitive awareness and individualised care [36]. Just as greater empowerment of women is associated with less violence, victims of violence are more willing to embrace ideals that empower women. Government commitment to promoting women’s empowerment would reduce violence against women and the associated costs [37].

The participants described how the specialised training for healthcare professionals may increase the detection of cases of violence against women and prevent iatrogenic interventions. Therefore, a key priority should be training healthcare professionals, given their crucial role in promoting awareness, trust, sensitivity, safety and confidentiality [18]. Our findings align with recent studies in Ecuador [38,39] which suggest that violence is not merely an individual event but a structural phenomenon. The ‘neutrality’ observed in some healthcare narratives may mask a form of symbolic violence, where the professional’s own immersion in a machoistic and religious social fabric leads to an unconscious normalization of gender-based aggression. This further highlights the urgent need for public policies that address early exposure to violence as a key factor, as well as the necessity of preventive measures focusing on educational programs and social interventions [39,40]. In Ecuador, evidence suggests that the inadequacy of existing healthcare protocols stems from a deep intertwining of cultural, social, and academic factors that normalize gender-based violence across both personal and institutional settings [40]. Beyond the clinical guidelines, the operational failure of these protocols is exacerbated by structural weaknesses, including chronic resource shortages, judicial bottlenecks, and a persistent lack of inter-institutional coordination, which prevents a seamless transition from healthcare detection to legal protection [41].

The healthcare professionals identified education as a key resource for combating violence against women. Therefore, greater emphasis should be place on their role in areas such as gender diversity, professional development and research [42]. Education contributes to a broader social understanding and as a means of addressing gender inequality. However, training for healthcare professionals is currently limited [43]. Sex education is essential for eliminating barriers, improving equality, preventing violence against women, and challenging false beliefs and sexism [44]. Likewise, health education helps to address emotional relationships between partners, recognise violence against women, and empower the individual to avoid adopting passive roles in situations of violence [45]. The narratives reveal internal ambivalences where professionals oscillate between their ethical duty and the internalization of the ‘patriarchal pact.’ This tension manifests as a professional contradiction: while advocating for empowerment, their discourse often slips into ‘neutrality’ or symbolic violence, reflecting the difficulty of challenging dominant social discourses. Acknowledging these zones of ambiguity is crucial, as it suggests that institutional change requires not only resources but also a reflexive ‘unlearning’ of the cultural reproduction of inequality by the practitioners themselves [41].

### 4.1. Strengths and Limitations of the Study

This study has several limitations that should be considered when interpreting the results. First, it was conducted in a single geographical area of Ecuador, which may limit the generalisability of the findings and conclusions. Second, a more representative sample would ideally include greater diversity in healthcare professions and professionals with experience in the legal processes related to violence against women. The multidisciplinary nature of the recruitment resulted in the underrepresentation of several healthcare fields. Specifically, the sample relied more heavily on professionals from psychology and neuroscience, whereas other essential roles—such as doctors and nurses—were represented by only two participants each. This imbalance may limit the depth of findings regarding the specific clinical experiences and unique perspectives inherent to each individual healthcare discipline. Notably, part of the sample had experienced violence against women, which allowed the victims’ perspectives to be incorporated. However, these experiences may also have affected their views, possibly leading to more negative interpretations.

### 4.2. Recommendations for Future Research

Future studies should move beyond descriptive designs toward participatory action research aimed at transforming institutional dynamics. There is ana specific and urgent need to evaluate the impact of integrating violence prevention and management courses directly into healthcare curricula, ensuring that future professionals develop early competencies in structural gender analysis. Furthermore, incorporating the dual perspectives of both victims and healthcare providers through longitudinal studies would provide critical data to refine these training programs, ensuring they address the real-world complexities of clinical care and the subjective needs of those experiencing violence.

### 4.3. Implications for Practice and Policy

Based on the identified institutional capacity gaps, policies should prioritize specialized training that moves beyond general awareness to address the specific lack of technical arguments reported by participants. Such training would enable professionals to transition from a reactive approach to a proactive identification of implicit violence, reducing the reliance on physical evidence alone. Furthermore, the findings suggest that updating action protocols is not enough; these protocols must explicitly incorporate strategies to avoid revictimization, directly responding to the professional ambivalence observed when practitioners face complex cases without clear institutional backing.

## 5. Conclusions

The healthcare professionals in this study emphasised the need to identify the key characteristics of violence against women, requiring attention to normalised forms of violence and to implicit, less visible expressions of abuse reflected in certain attitudes and behaviours. The participants perceived that the current social context and deep-rooted power dynamics favour the perpetuation of violence against women. Therefore, findings suggest that prevention strategies in Ecuador should prioritize early detection protocols, interdisciplinary coordination, and structured gender-sensitive training for healthcare professionals to prevent and eradicate violence against women. This training should be reformulated to include practical knowledge of healthcare and judicial procedures, formal reporting pathways, and the specific community support resources available to victims. By addressing these academic deficiencies, professionals can bridge the gap between legal theory and daily clinical practice, ensuring a more effective institutional response. Moreover, healthcare professionals can play an important role in the prevention of violence against women by improving care for survivors, identifying areas for improvement within existing prevention systems, and promoting sex education. Beyond the identification of normalized violence and power dynamics, this study reveals a critical tension in professional praxis characterized by the persistent conflict between structural inequality awareness and the concrete limitations of specific institutional frameworks.

## Figures and Tables

**Table 1:** Sociodemographic data of the participants.

Participant	Sex	Age	Marital Status	Children	Religion	Profession	Work Experience (Years)
1	Female	58	Married	1 son 2 daughters	Catholic	Clinical psychologist	13 years
2	Female	47	Married	1 son 1 daughter	Catholic	PHD in Neuroscience	19 years
3	Female	46	Married	3 daughters	Catholic	PHD in Education	17 years
4	Male	55	Married	2 daughters 1 son	Catholic	Doctor	23 years
5	Female	30	Married	1 daughter	Catholic	PHD in Neurodevelopment	5 years
6	Female	35	Separated	1 son	Evangelical	Doctor	9 years
7	Female	31	Married	2 sons	Jewish	Educational-therapeutic psychologist	11 years
8	Female	42	Married	2 sons	Catholic	Nurse	15 years
9	Male	37	Married	1 daughter 1 son	Catholic	Social worker	8 years
10	Female	52	Divorced	2 daughters	Catholic	Nurse	24 years

**Table 2:** Interview protocol.

Stage	Title	Content/Example Questions
Introduction	Purpose	The perceptions of healthcare professionals provide information that should be known to all.
	Objectives	Understanding the perceptions of healthcare professionals regarding violence against women.
Start	General introductory question	Perhaps we could start with you telling me what the term “violence against women” means to you.
Development	‘What does the term “tip of the iceberg” suggest to you in terms of violence against women?’ ‘What discriminatory behaviours do you think generally lead to violence?’ ‘How could violence against women be identified in a victim? What should we look for first?’	
Cierre	Final questions	Do you think I have left any questions unanswered, anything you would like to add?
	Acknowledgements	Thank you very much for your time, it has been very valuable. Many important issues will arise from this and please be aware that your contribution will be of great help to us.

**Table 3:** Themes, subthemes and units of meaning.

Theme	Subtheme	Units of Meaning
The characteristics and identification of violence against women.	Normalisation of microaggressions as structural violence	Gender-based microaggressions, non-specific symptoms.
	At the bottom of the iceberg: identifying implicit violence	Subtle violence, normalising discriminatory behaviour, unconscious perpetuation, avoid revictimization
The social dimensions of violence against women.	The social factors underpinning the persistence of violence against women	Structural gender asymmetry, traditional gender role socialization, sexist religious dogmatism
	Power dynamics that encourage violence against women	Social difficulties, social privileges, opportunities or rights
	Social approaches to combating violence against women	Training of professionals, reviewing protocols, socio-economic empowerment.
Combating violence against women in clinical and educational settings.	The duty of professionals in addressing violence against women	Personalised approach, multidisciplinary perspective, institutional capacity gaps
	Identifying systemic failures in the eradication of violence against women	Questioning a victim’s testimony, lack of human resources, lack of financial resources
	Promoting sex education	Education in schools, health education, removing barriers

## Data Availability

The data generated and analysed in the study are not publicly available due to the privacy and confidentiality agreement with the participants. They are available upon request to the corresponding author.

## Supplementary Materials

The following supporting information can be downloaded at: https://www.mdpi.com/article/10.3390/healthcare14091146/s1, Table S1: COREQ (Consolidated Criteria for Reporting Qualitative Research) Checklist. Ref. [24] is cited in Supplementary Materials.
