# Supplementary material for: The Perceptions of Healthcare Professionals Regarding Violence Against Women in Ecuador: A Qualitative Study
Source: Healthcare (Basel). 2026 Apr 24;14(9):1146. doi: 10.3390/healthcare14091146 (PMC13163525; doi:10.3390/healthcare14091146)
Supplement: Supplementary file 1 [file healthcare-14-01146-s001.zip › healthcare-4186588-supplementary.pdf]

Manuscript: *The perceptions of healthcare professionals regarding violence against women: a qualitative study.*

Consolidated criteria for reporting qualitative studies (COREQ): 32-item checklist

Reference: Tong A, Sainsbury P, Craig J. Consolidated criteria for reporting qualitative research (COREQ): a 32-item checklist for interviews and focus groups. *International Journal for Quality in Health Care*. 2007. Volume 19, Number 6: pp. 34.

DOMAIN AND NUMBER OF ÍTEM	GUIDE QUESTIONS/DESCRIPTION	REPORTED ON PAGE
**Domain 1: Research team and reflexivity**		
**Personal Characteristics**		
1. Interviewer/facilitator	Which author/s conducted the interview or focus group?	Three Ecuadorian researchers p. 4 line 121
2. Credentials	What were the researcher´s credentials? E.G. PhD, MD	Title page (Ph.D.)
3. Occupation	What was their occupation at the time of the study?	Title page
4. Gender	Was the researcher male or female?	Title page. P. 4: Interviews: three women researchers
5. Experience and training	What experience or training did the researcher have?	p. 4 (data collection)
**Relationship with participants**		
6. Relationship established	What did the participants know about the researcher? e.g. personal goals.	Table 2. Interview protocol (p. 4)
7. Participant knowledge of the interviewer	What did the participants know about the researcher?	Table 2. Interview protocol (p.4)
8. Interviewer characteristics	What characteristics were reported about the inter viewer/facilitator?	p. 4, data collection
**Domain 2: study design**		
**Theoretical framework**		
9. Methodological orientation and Theory	What methodological orientation was stated to underpin the study?	p. 3. Design
**Participant selection**		
10. Sampling	How were participants selected?	p. 3. Participants and context
11. Method of approach	How were participants approached?	p. 3. Participants and context
12. Sample size	How many participants were in the study?	p. 3. Participants and context
13. Non-participation	How many people refused to participate or dropped out? Reasons?	p. 3. Participants and context
**Setting**		
14. Setting of data collection	Where was the data collected?	p. 3. Participants and context
15. Presence of nonparticipants	Was anyone else present besides the participants and researchers?	p. 3. Participants and context
16. Description of sample	What are the important characteristics of the simple?	p. 3. Table 1
**Data collection**		

17. Interview guide	Were questions, prompts, guides provided by the authors? Was it pilot tested?	P. 4, Table 2
18. Repeat interviews	Were repeat inter views carried out? If yes, how many?	A single interview (P. 4)
19. Audio/visual recording	Did the research use audio or visual recording to collect the data?	P. 4. Data collection
20. Field notes	Were field notes made during and/or after the interview or focus group?	P. 4. Data collection
21. Duration	What was the duration of the inter views or focus group?	P. 4. Data collection
22. Data saturation	Was data saturation discussed?	P. 4. Data collection
23. Transcripts returned	Were transcripts returned to participants for comment and/or correction?	P. 5. Rigour
**Domain 3: analysis and findings**		
**Data analysis**		
24. Number of data coders	How many data coders coded the data?	Rigor
25. Description of the coding tree	Did authors provide a description of the coding tree?	Table 3
26. Derivation of themes	Were themes identified in advance or derived from the data?	Results
27. Software	What software, if applicable, was used to manage the data?	ATLAS.ti (p. 4, Data analysis
28. Participant checking	Did participants provide feedback on the findings?	P. 5. Rigour
**Reporting**		
29. Quotations Presented	Were participant quotations presented to illustrate the themes/findings? Was each quotation identified? e.g. participant number	Pages 6-14, Results
30. Data and findings consistent	Was there consistency between the data presented and the findings?	Pages 6-14, Results
31. Clarity of major themes	Were major themes clearly presented in the findings?	Table 3 and pages 6-14, Results
32. Clarity of minor themes	Is there a description of diverse cases or discussion of minor themes?	Table 3 and pages 6-14, Results
